# Case Report: Pulmonary arterial hypertension in children caused by a new mutation in the *BMPR2* gene

**DOI:** 10.3389/fped.2025.1572733

**Published:** 2025-05-27

**Authors:** Ting Tang, Shuqi Wu, Chang Peng, Li Wang

**Affiliations:** Department of Pediatrics, Guizhou Children’s Hospital, Affiliated Hospital of Zunyi Medical University, Zunyi, Guizhou, China

**Keywords:** pulmonary arterial hypertension, *BMPR2* mutation, deletion insertion mutation, children, diagnosis

## Abstract

Pulmonary arterial hypertension (PAH) is a rare and severe condition that has been linked to hereditary factors. Mutations in the gene encoding bone morphogenetic protein receptor 2 (*BMPR2*) have been identified as a cause of heritable PAH. We report the discovery of a novel point mutation combined with a deletion insertion mutation (c.621+2T>C/c.621+5_621+11delinsA) in the *BMPR2* gene of an 11-year-old PAH patient lacking a family history of genetic disease (Clinical trial number: not applicable). This report expands the genetic landscape and offers a scientific foundation for early disease detection, personalized treatment strategies, and genetic counseling.

## 1 Introduction

Pulmonary arterial hypertension (PAH) is a cluster of diseases marked by a relentless rise in pulmonary vascular resistance and progressive right heart failure, which can be triggered by mutations in specific genes (1). The bone morphogenetic protein receptor 2 (*BMPR2*) gene, which codes for a protein known as bone morphogenetic protein receptor II, crucial for sustaining pulmonary vascular equilibrium, is mutated in approximately 75% of cases of heritable pulmonary arterial hypertension (HPAH) (2). When the *BMPR2* gene is altered, it initiates a cascade of pathological changes, including vasoconstriction, heightened inflammatory responses, endothelial cell dysfunction, and eventually PAH (3). PAH in pediatric patients constitutes a unique clinical syndrome characterized by a more rapid progression of the disease compared to adult-onset cases. Genetic susceptibility is particularly significant in this context. More than 70% of heritable PAH cases and approximately 20% of idiopathic pediatric PAH cases are associated with pathogenic variants in the *BMPR2* gene, which predominantly consist of loss-of-function mutations that disrupt the signaling pathways of bone morphogenetic proteins (BMPs). Recent research highlights the profound clinical implications of *BMPR2* mutations within pediatric populations: individuals with these mutations present symptoms 5.8 years earlier than those without (median age 6.4 vs. 12.2 years), and they face a 3.2-fold increased risk of right ventricular failure within the first two years post-diagnosis (4, 5). The relationship between genotype and phenotype also influences therapeutic outcomes, as patients with *BMPR2* deficiency exhibit diminished vasoreactivity to nitric oxide and necessitate an earlier transition to prostacyclin analog therapy (6). PAH is a rare yet severe condition that truncates life expectancy. Consequently, the early identification, diagnosis, and treatment of PAH can significantly enhance the prognosis of this potentially fatal disease (7). In this report, we present the case of a boy who was admitted to the Department of Pediatric Respiratory and Cardiovascular Medicine at our hospital and diagnosed with PAH due to a mutation in the *BMPR2* gene. Whole-exome testing revealed a novel mutation site (c.621+2T>C/c.621+5_621+11delinsA) within the *BMPR2* gene, and the clinical data are detailed herein.

## 2 Clinical data

The patient, a 11-year-old male, was admitted to the hospital due to recurrent episodes of wheezing and shortness of breath lasting over three months, accompanied by a ten-day history of coughing, panic, palpitations, and chest tightness. The child began experiencing wheezing and shortness of breath following an early morning jog more than three months ago, accompanied by chest discomfort, palpitations, occasional dizziness, pallor, and profuse sweating. These symptoms have been alleviated by rest over the past three months. Over the last three months, the child has exhibited symptoms of wheezing and shortness of breath, particularly subsequent to ascending a flight of stairs for approximately three minutes. This is accompanied by palpitations, chest tightness, and sweating, all of which alleviate with rest. The child was admitted to another hospital for further treatment 10 days ago after developing a cough, which was characterized by monosyllabic sounds and white sputum.

The patient's history, encompassing drug and solvent exposure, as well as comprehensive clinical, serologic, laboratory, and imaging assessments, did not indicate any connective tissue diseases, portal hypertension, human immunodeficiency virus infection, or other conditions linked to pulmonary hypertension. Notably, the patient had undergone “repair of ventricular septal defect” at seven months of age, yet no pulmonary hypertension or residual shunt was detected during the five-year postoperative follow-up. There was no history of infectious diseases such as hepatitis or tuberculosis. No history of food or drug allergies was reported. The patient denied any history of blood transfusions. The patient born full-term via Cesarean section, with no history of asphyxia or resuscitation, and no history of mechanical ventilation or exposure to high oxygen concentrations. The birth history, feeding history, and developmental history were all unremarkable. There were no instances of consanguineous marriages in the family tree, both parents were previously healthy, and there was no history of inherited metabolic diseases. However, the patient's grandfather's brother had a history of cardiac disease diagnosed during his adolescence within the family, though the specific diagnosis remains unclear.

Laboratory tests conducted at our hospital indicated the following for the myocardial infarction biomarkers: myoglobin at 21 ng/ml, high-sensitivity troponin T at 20.4 ng/L, and N-terminal pro-B-type natriuretic peptide (NT-proBNP) at 2,477 pg/ml. Echocardiography revealed an aneurysmal dilatation of the pulmonary artery, severe pulmonary hypertension, and postoperative repair of a ventricular septal defect with no atrial, ventricular, or arterial shunting. There was also evidence of right heart enlargement and bilobed pulmonary valve malformations, with an estimated pulmonary systolic pressure of approximately 133 mmHg (normal reference range: 15–30 mmHg) based on tricuspid regurgitation. The left ventricular ejection fraction was 69% (normal reference range: 55%–70%), and the fractional shortening was 39% (normal reference range: 25%–45%). The electrocardiogram showed sinus tachycardia and right ventricular hypertrophy with strain. Considering that cardiac catheterization is an invasive procedure and there are ethical concerns associated with it, it is not the primary examination for pediatric patients. Therefore, this examination was not carried out on this pediatric patient.

**Figure 1:**
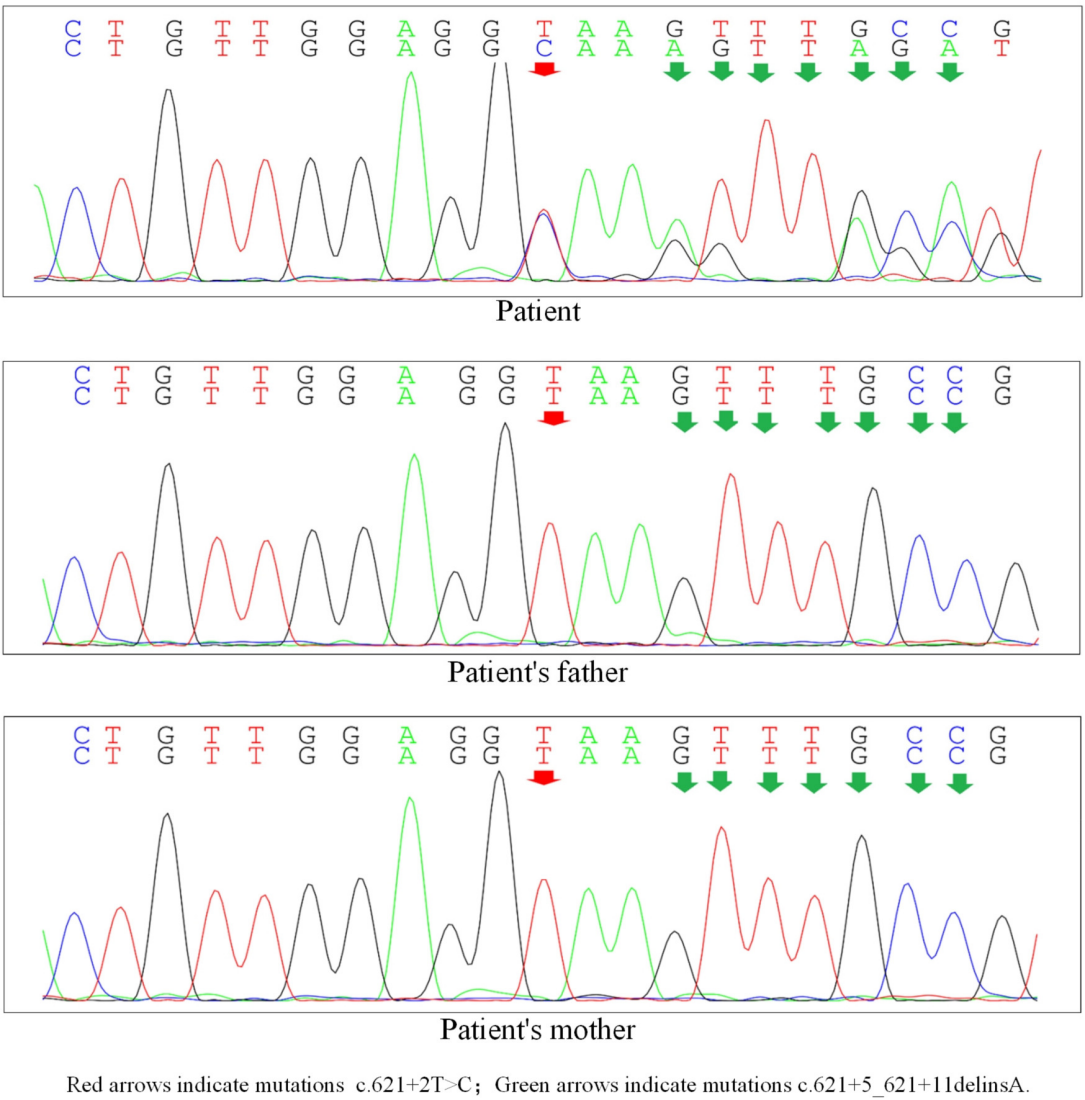
Gene sequencing map (transcript version number: NM_LC24052700033001AA1).

Upon obtaining informed consent, blood samples were collected from the patient and both parents for whole-exome sequencing (100 × coverage) and mitochondrial genome sequencing (3,000 × coverage) (8). The genetic analysis revealed minor variants (Single Nucleotide Variants/Insertions and Deletions) (SNVs/InDels) in the BMPR2 gene: c.621+2T>C/c.621+5_621+11delinsA, which were classified as pathogenic by ACMG classification (American College of Medical Genetics and Genomics) (Clinical trial number: not applicable).These variants are linked to familial primary pulmonary hypertension (type I), with or without hereditary hemorrhagic telangiectasia, an autosomal dominant disorder. As shown in Figure 1 and Table 1. Particularly, the mutations c.621+2T>C and c.621+5_621+11delinsA, situated within the intronic region, may influence the gene's structure and function. Such changes could lead to alterations in the transcribed RNA, which in turn affects protein synthesis, potentially disrupting essential biological processes including cell growth, differentiation, and metabolism (9). Furthermore, we employed four distinct software tools to forecast the impact of these alterations on donor/acceptor splice sites. Within the SPIDEX software's predictive model, a positive score signifies the enhancement of splicing, whereas a negative score signifies its inhibition. The magnitude of the absolute value correlates with the variant's influence on splicing; the greater the absolute value, the more pronounced the effect. A score of −15.26 suggests that the genetic variant is likely to exert a significant negative impact on splicing, potentially leading to diminished splicing efficiency or the emergence of aberrant splicing events. In the context of SpliceAI's predictive analysis, scores of 0.04 and 0.09 suggest that the variant might marginally improve the recognition of splice sites, albeit with a negligible effect. The MutationTaster software's prediction, denoted by the term “Disease causing”, implies that the variant could impair gene function, thereby possibly precipitating disease onset. The number 1 signifies the prediction's confidence level, with lower numbers reflecting greater confidence in the prediction. Consequently, the variant is strongly suspected to be disease-inducing. Lastly, the Genomic Evolutionary Rate Profiling (GERP) software's prediction, marked by “Conserved”, indicates that the gene's position is conserved across various species, signifying minimal evolutionary change. A score of 5.92 suggests that the higher the score, the more conserved the position is, and it may be crucial for biological functionality (10). As illustrated in Table 2.

**Table 1:** Sequencing verification results in *BMPR2* gene mutation.

Gene	Chromosomal location	Nucleotide varia	Intron	Genotype	ACMG rating
*BMPR2*	chr2:203379704	c.621+2T>C	intron5	Heterozygous	Suspected pathogenic
*BMPR2*	chr2:203379707	c.262+5_621+1ldelinsA	intron5	Heterozygous	Unclear clinical significance

A, adenine; *BMPR2*, bone morphogenic protein receptor type2; chr, chromosome; C, cytosine; T, thymine.

**Table 2:** Prediction of protein functional damage in *BMPR2* gene mutation.

Nucleotide variant	Prediction software	Score
c.621+2T>C	SPIDEX	−15.26
	Splice AI	0.04
	Mutation taster	Disease causing (1)
	GERP	Conserved (5.92)
c.621+5_621+11delinsA	Splice AI	0.09

A, adenine; C, cytosine; T, thymine; SPTDEX, splicing-based predictor for individualized exon vsage; Splice AI, spling artificial intelligence; GERP, genomic evolutionary rate profiling.

## 3 Discussion

PAH, a cluster of disorders, is marked by symptoms such as breathlessness, fatigue, and fainting (11). It is characterized by a significant dilation of the pulmonary vasculature's internal diameter, elevated pulmonary artery pressure, and hypertrophy of the right ventricle (12). The pathophysiological alterations impede the normal antegrade movement of blood within the pulmonary circulation. As a result, persistent disruptions in pulmonary hemodynamics can culminate in the emergence of right-heart failure, via mechanisms including heightened pulmonary vascular resistance leading to augmented right-ventricular afterload, right-ventricular hypertrophy, and subsequent decompensation (13). In recent times, a growing body of research has concentrated on the established fact that mutations in *BMPR2* are associated with pathogenicity in PAH (14). The identified genes responsible include Neurogenic locus notch homolog protein 3 (*NOTCH3*), *BMPR2*, Actin Alpha 2, Smooth Muscle (*ACTA2*), Vascular Endothelial Growth Factor Receptor 2 (*VEGFR2*), T-box transcription factor 4 (*TBX4*), and SRY-box transcription factor 17 (*SOX17*), which facilitate the emergence and advancement of pulmonary hypertension via distinct mechanisms (15). The protein produced by the *BMPR2* gene, as detailed in this report, is a pivotal element of the bone morphogenetic proteins (BMPs) signaling pathway, playing a crucial role in the formation of the cardiovascular system during embryonic stages (16). In individuals with PAH, mutations in the *BMPR2* gene disrupt the normal BMPs signaling, thereby impacting the proper development and upkeep of the pulmonary vasculature (17). The *BMPR2* protein also operates within the transforming growth factor β (TGF-β) pathway. It regulates the phosphorylation of downstream Smad proteins, which are pivotal mediators in the TGF-β signaling cascade (18). The protein influences various cellular responses, including cell proliferation, differentiation, and apoptosis, within the context of pulmonary arterial hypertension pathogenesis. By interacting with non-BMP-specific elements of the TGF-β pathway, the *BMPR2* protein contributes to the overall complexity of the molecular mechanisms underlying the disease (19). These mutations encompass a spectrum of types, such as missense mutations, nonsense mutations, and splice site variants, all of which can result in defective *BMPR2* protein function. This dysfunction can subsequently lead to heightened pulmonary arterial pressure and structural anomalies within the tissues (20).

In this study, we identified novel intronic shear mutation sites and deletion and insertion mutations (c.621+2T>C, c.621+5_621+11delinsA) in the *BMPR2* gene. Numerous mutations previously associated with pulmonary hypertension include nonsense mutations (c.631C>T, c.76C>T; p.Gln26Ter), missense mutations (c.604A>T, c.794A>G, c.797G>C, and c.806G>T), shear mutations (c.852+1G>C, c.853-1G>C, c.621+1G>A), promoter deletions and insertion mutations (c.621+5_621+11delinsA), promoter mutations (c.-575A>3ET, c.-586dupT), and shift mutations (c.612del A, c.690-691del AGins T, and c.673-679del CGTCCAG). In this report, we have found that the mutation of 621+2T>C, an intron, adheres to the GT-AG rule. The GT-AG rule is essential for the splicing of eukaryotic pre-mRNA, wherein introns are delineated by the presence of GT (or GU in RNA) at the 5′ terminus and AG at the 3′ terminus. The spliceosome employs these specific sequences to facilitate the processing of pre-mRNA into mature mRNA, which is subsequently translated. In the course of our investigation into *BMPR2* mutations, we hypothesize that the mutation impacts the GT-AG splicing sites within the *BMPR2* gene. Given the pivotal role of *BMPR2* in the TGF-β signaling pathway, which is instrumental in the regulation of pulmonary arterial cells, the resultant mutant protein diminishes the phosphorylation of downstream small mothers against decapentaplegic (SMADs) (21). This impairment disrupts the signaling cascade, leading to aberrant cellular behaviors such as unchecked proliferation and resistance to apoptosis, which are characteristic of heritable pulmonary arterial hypertension (PAH) (18). Although this mutation does not directly alter the protein's amino acid sequence, it indirectly affects gene expression and protein function by disrupting the gene splicing process. The post-shear insertion change of c.621+5_621+11delinsA may impact the structure and function of the gene (22). We believe that the incomplete sequence of the *BMPR2* gene could lead to defective *BMPR2* function. We correlated the features of our pediatric patient with established *BMPR2*-related PAH phenotypes from the literature. The patient's early onset, severe echocardiographic findings, treatment response, and nuances of family history, along with the presence of pulmonary artery aneurysmal dilation, align with known patterns of *BMPR2*-associated PAH (23). For long-term care, we adhered to the guidelines of the American College of Chest Physicians (ACCP) and incorporated genetic counseling for *BMPR2* mutation carriers. This includes emphasizing autosomal-dominant inheritance, suggesting cascade testing for first-degree relatives, regular cardiopulmonary screening, and specific monitoring for asymptomatic carriers. The discovery of the mutation informs personalized prevention, drug selection, and early transplantation evaluation. However, since pathogenicity relies on bioinformatics, we will communicate its limitations during counseling and integrate relatives’ test results with clinical phenotypes for ongoing risk assessment of asymptomatic individuals (24).

PAH, a condition that not only endangers patients' health but also imposes significant psychological and financial strains on their families and society, has long been a formidable challenge for the medical community due to its intricate and hereditary characteristics (9). The genetic complexity of PAH means that the identification of causative genes is an ongoing process necessitating advanced genomic research (25). Research has indicated that individuals with PAH and *BMPR2* mutations tend to experience more severe symptoms at a younger age and face a heightened mortality risk compared to those without such mutations (26). Recent studies reveal that 10%–15% of childhood-onset PAH cases are caused by *de novo* (newly occurring) *BMPR2* mutations, even without family history (27). Consequently, genetic testing is especially crucial for PAH patients. This report uncovers a novel mutation locus, which will facilitate future clinical research and the identification of new therapeutic targets (28). The genetic tests detailed in this report can serve as a guide for clinicians in early detection and intervention, potentially enhancing the quality of life and prognosis for patients. Considering that this investigation constitutes the inaugural identification of the novel *BMPR2* mutation, the proposed applications within these domains are speculative in nature. They delineate potential avenues for subsequent research endeavors. It is explicitly stated that these concepts necessitate further scrutiny and validation. For example, cohort-based investigations are indispensable for establishing dependable biomarkers for early detection, and meticulously designed clinical trials are imperative to explore personalized treatment modalities. The recently identified *BMPR2* mutation, despite its comprehensive implications being unverified, may influence early disease detection, personalized treatment approaches, and genetic counseling (29). In the context of early detection, it could potentially steer the discovery of biomarkers, where alterations in pertinent protein or metabolite levels can be utilized to accurately evaluate treatment efficacy and modify regimens as required. For personalized treatment, it might inform the selection of drugs targeting the affected pathways. In response to the condition of the pediatric patient in this instance, medications such as sildenafil and propranolol were administered during the hospitalization period. During the subsequent telephone follow - up, not only did the pediatric patient not exhibit any significant adverse reactions to the medications, but also clear signs of improvement became evident. The shortness of breath, which was previously limiting the patient's activity, was markedly reduced. The patient could now engage in light physical activities without experiencing the same degree of breathlessness. Symptoms such as chest discomfort, palpitations, and dizziness have all been alleviated to varying degrees, indicating that the treatment was efficacious. With respect to genetic counseling, it augments the knowledge for predicting inheritance patterns and guiding family-based testing, thus holding significance in these critical areas. Nevertheless, the report acknowledges certain limitations, including insufficient data, recall bias, restricted study scope, and limited practical application. It is imperative to acknowledge the constraints inherent in this singular case study. The primary disadvantage is the diminished statistical power resulting from the limited sample size. This limitation impedes our capacity to generalize the observed outcomes to the larger population of individuals with heritable PAH. It is essential to note that due to unforeseen and uncontrollable factors, we lack a few perspectives from the patient and their family. Notwithstanding these constraints, the thorough examination of this specific case has yielded distinctive insights that may function as a foundation for additional research endeavors. With the continuous advancement of gene editing technology and bioinformatics, we anticipate offering more effective and safer treatment options for PAH patients, ultimately aiming to achieve long-term management or even a cure for the disease.

## Data Availability

The datasets presented in this study can be found in online repositories. The names of the repository/repositories and accession number(s) can be found in the article/Supplementary Material.
